# Seizures elevate gliovascular unit Ca^2+^ and cause sustained vasoconstriction

**DOI:** 10.1172/jci.insight.136469

**Published:** 2020-10-02

**Authors:** Cam Ha T. Tran, Antis G. George, G. Campbell Teskey, Grant R. Gordon

**Affiliations:** 1 Hotchkiss Brain Institute and; 2 Department of Physiology and Pharmacology, Cumming School of Medicine, University of Calgary, Calgary, Alberta, Canada.; 3 Department of Physiology and Cell Biology, University of Nevada, Reno School of Medicine, Reno, Nevada, USA.; 4 Department of Cell Biology and Anatomy, Cumming School of Medicine, University of Calgary, Calgary, Alberta, Canada.

**Keywords:** Neuroscience, Epilepsy, Neuroimaging, Seizures

## Abstract

Seizures can result in a severe hypoperfusion/hypoxic attack that causes postictal memory and behavioral impairments. However, neither postictal changes to microvasculature nor Ca^2+^ changes in key cell types controlling blood perfusion have been visualized in vivo, leaving essential components of the underlying cellular mechanisms unclear. Here, we use 2-photon microvascular and Ca^2+^ imaging in awake mice to show that seizures result in a robust vasoconstriction of cortical penetrating arterioles, which temporally mirrors the prolonged postictal hypoxia. The vascular effect was dependent on cyclooxygenase 2, as pretreatment with ibuprofen prevented postictal vasoconstriction. Moreover, seizures caused a rapid elevation in astrocyte endfoot Ca^2+^ that was confined to the seizure period, and vascular smooth muscle cells displayed a significant increase in Ca^2+^ both during and following seizures, lasting up to 75 minutes. Our data show enduring postictal vasoconstriction and temporal activities of 2 cell types within the neurovascular unit that are associated with seizure-induced hypoperfusion/hypoxia. These findings support prevention of this event may be a novel and tractable treatment strategy in patients with epilepsy who experience extended postseizure impairments.

## Abstract 2

Seizures cause enduring microvascular constriction via long lasting calcium elevations in vascular smooth muscle cells.

## Graphical abstract

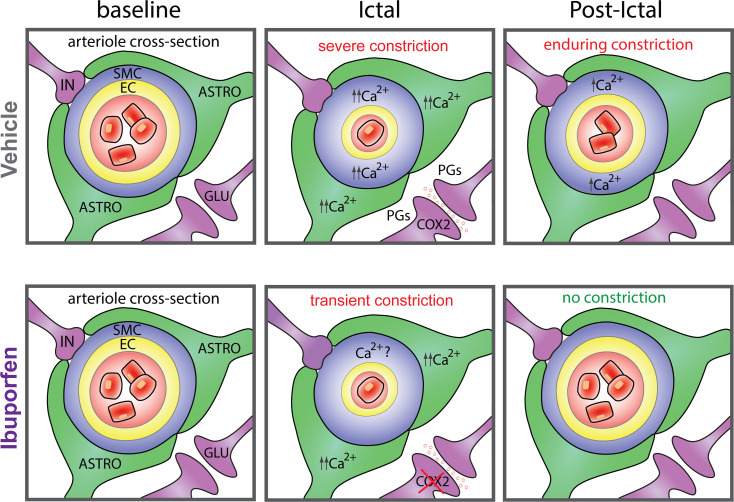

## Introduction

Following seizure termination, motor, sensory, and/or memory impairments can be expressed, and each symptom is thought to relate to the specific brain structures participating in the seizure (1, 2). A notable example is Todd’s paresis, which specifically pertains to moderate to severe motor weakness following seizures and usually subsides within a few hours (3). Because the symptoms are similar to ischemic stroke, Todd’s paresis is often misdiagnosed (4, 5). Farrell et al. systematically investigated local oxygen levels and blood flow following evoked or self-generated seizures in behaving rodents and discovered a severe hypoxic event (partial pressure of oxygen in mmHg [pO_2_] < 10 mmHg) that lasted for more than 1 hour. This phenomenon was the result of blood hypoperfusion and it generalized to people with epilepsy (6, 7). Previous studies have shown that brain region–specific postictal memory and behavioral impairments were caused by this hypoperfusion/hypoxic event (6). Therefore, seizures can result in a stroke-like attack, which is responsible for the postictal state and may also be implicated in chronic behavioral comorbidities and anatomical alterations associated with epilepsy (8).

Cyclooxygenase 2 (COX-2) and L-type Ca^2+^ channel activity during seizures were identified as key mechanisms necessary for the precipitous drop in tissue oxygen (6). Use of those target inhibitors showed the postictal hypoxia block was downstream of epileptiform neural activity, pointing to direct actions on contractile components of the neurovascular unit responsible for controlling blood perfusion. However, previously employed oxygen-sensitive probes and laser Doppler flowmetry did not provide any information on cell type activities underlying the phenomenon (e.g., vascular smooth muscle cells [VSMCs] that coat penetrating and pial arterioles) (9, 10). VSMCs are the end effectors that control arteriole diameter and make a significant contribution to the regulation of microcirculatory blood flow and cortical oxygenation (11, 12). VSMC membrane potential, coupling to L-type Ca^2+^ entry for excitation-contraction coupling, is a central mechanism mediating changes to arteriole tone. However, Ca^2+^-independent mechanisms of vasoconstriction also exist (13, 14), including pathological vasospasms (15); therefore, it is important to measure VSMC Ca^2+^ levels during and after a seizure to corroborate or refute a vascular Ca^2+^ hypothesis. Previous amelioration of postictal hypoxia with nifedipine argues in favor for a contribution from VSMC free Ca^2+^ (6). Additionally, astrocytes are important players in the regulation of local brain blood flow via their perivascular endfeet, which communicate to VSMCs. Astrocytes can release a number of vasodilator or vasoconstrictor messengers in a Ca^2+^-dependent manner (16, 17). Although there is little doubt that a large, transient increase in endfoot Ca^2+^ can control arteriole diameter (18–21), recent studies have shown that endfeet can undergo long-lasting changes to free Ca^2+^ in response to neural activity, which translate into enduring increases in steady-state arteriole tone (22). Additionally, quantitative elevations in astrocyte endfeet Ca^2+^ correlate with microvascular diameter changes during epileptiform activity using the 4-aminopyridine seizure model in anesthetized mice (23). However, examining Ca^2+^ signals in astrocyte endfeet and VSMCs in awake mice, rather than in brain slices or in vivo preparations using anesthesia or sedation, is important because signaling pathways involving neurons, astrocytes, and the microvasculature can crosstalk realistically. Thus, imaging behaving mice is essential to prevent misrepresentation of the relationships between hypersynchronous neural activity, astrocyte and VSMC Ca^2+^, and hemodynamics (24–26). Here, we performed in vivo 2-photon imaging on awake head-restrained mice to examine Ca^2+^ activity patterns of cortical astrocytes and VSMCs during ictal and postictal periods to gain insight into the cellular underpinnings of seizure-induced hypoperfusion/hypoxia.

## Results

### Long-lasting hypoxia follows maximal electroconvulsive seizures in awake freely moving mice and generalizes across sexes and Cre-lox lines.

**Figure 1:**
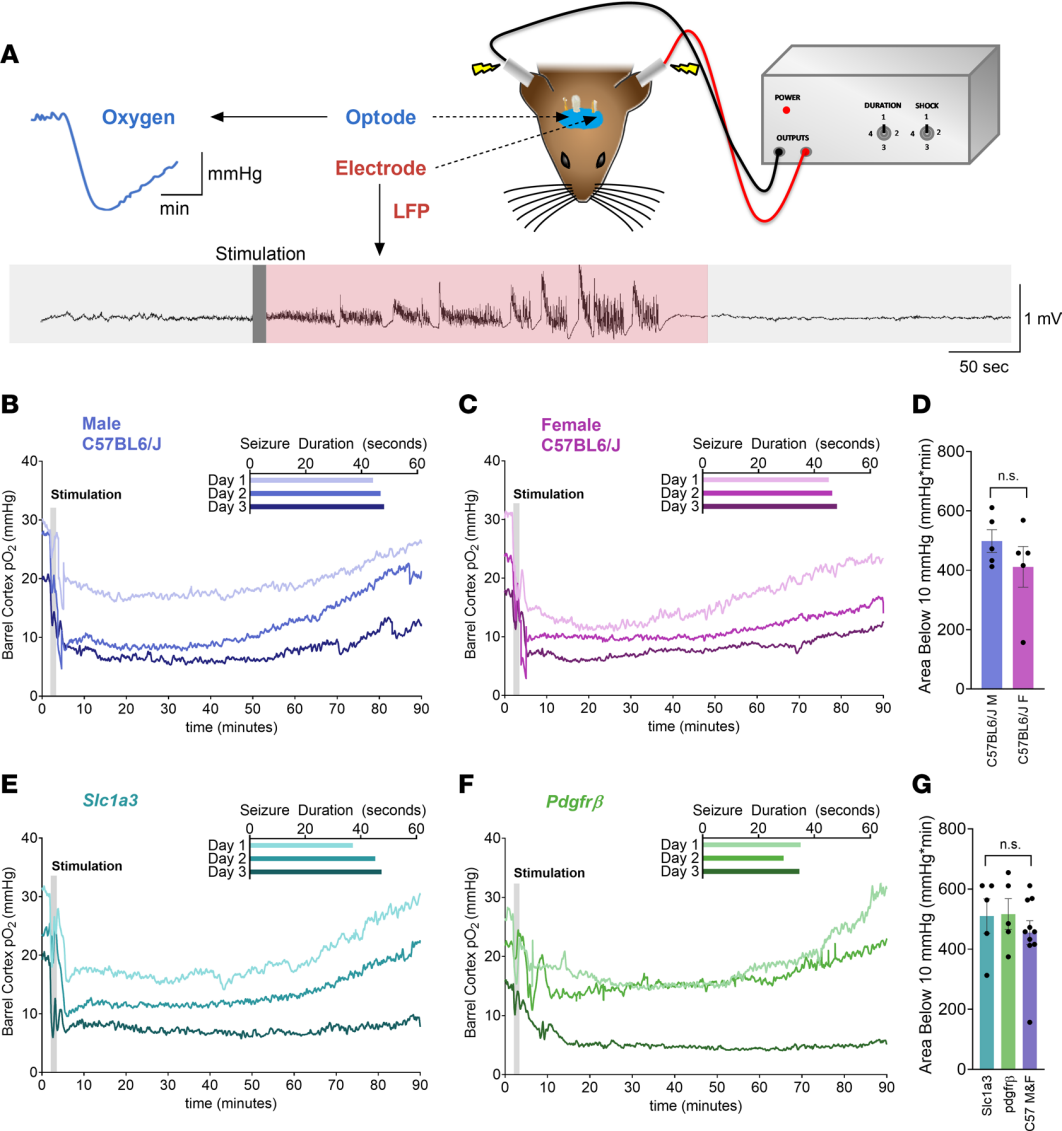
MES seizures induce postictal hypoxia that generalizes across sex and strains. (**A**) Experimental setup has mice with a chronically implanted optode and electrode in their barrel cortex. Awake freely moving mice received one 0.2-second MES per day for 3 days with concurrent local field potential and local partial pressure of oxygen (pO_2_) recordings. (**B** and **C**) Mean oxygen profile before, during, and after MES in male (**B**) and female (**C**) C57BL/6J mice (*N* = 5, each). The inset shows the duration of electrographic seizures during the 3 seizures. (**D**) Quantification (mean ± SEM) of the area (depth and duration) below the severe hypoxic threshold (pO_2_ < 10 mmHg) following the third seizure. Male and female C57BL/6J mice were not different from each other (*t* test, t[8] = 1.11, *P* = 0.30). (**E** and **F**) Mean oxygen profile before, during, and after MES in mixed sex *Slc1a3* (astrocyte reporter) (**E**) and *PdgfrB* (mural cell reporter) (**F**) mice (*N* = 5, each). (**G**) Quantification (mean ± SEM) of the area below the severe hypoxic threshold (pO_2_ < 10 mmHg) following the third seizure. *Slc1a3* and *PdgfrB* and combined male and female C57BL/6J mice were not different from each other (1-way ANOVA, F[2,17] = 0.56, *P* = 0.58).

Seizure-induced hypoxia was previously demonstrated using several animal models of epilepsy (6). However, to examine postictal hypoxia in head-restrained active mice under the 2-photon microscope, we employed the maximal electroconvulsive shock (MES) technique (27) because it evokes time-locked, widespread seizure activity across the neocortex (28), which can be captured at any arbitrary penetrating arteriole. Before imaging, we first validated MES-induced hypoxia in male (*N* = 5) and female (*N* = 5) C57BL/6 mice that were awake and freely moving (no head restraint). Local field potentials and pO_2_ were chronically monitored in layer V of mouse barrel neocortex during 3 brief, discrete seizures (Figure 1A). A total of 200 ms of MES reliably manifested hypersynchronous epileptiform activity (Figure 1A) for 32–57 seconds for male mice and 33–55 seconds for female mice (Figure 1, B and C inset), which produced tonic-clonic behavioral seizures analogous to generalized bilateral seizures in people with epilepsy. The mean baseline pO_2_ in the barrel cortex of freely moving mice over the 2 days of recordings ranged between 20 and 30 mmHg in male mice and 18 and 32 mmHg in female mice (*P* > 0.05) (Figure 1, B and C). After each seizure, oxygen levels fell rapidly, taking more than 1 hour to recover to baseline levels. Following the third seizure, a sustained decrease in pO_2_ was observed that was below the severe hypoxic threshold of 10 mmHg (Figure 1, B and C). This threshold is defined with a molecular, cellular, and clinical signature (29–33); therefore, we quantified the integral (area) of the pO_2_ curve below this oxygen level (6). Because no significant differences in seizure duration and postictal hypoxia were observed between the 2 sexes (Figure 1D), for subsequent experiments we mixed both sexes and pooled the data. Next, we tested for similar MES-induced hypoxia in the knockin Cre-lox crosses planned for 2-photon fluorescence microscopy. First, postictal hypoxia was examined in *Slc1a3*-Cre/ERT × RCL GCaMP3 mice (*N* = 5), which permit visualization of astrocyte free Ca^2+^ using the excitatory amino acid transporter 1 promoter (GLAST) (34). Second, we examined *PdgfrB*-Cre × RCL-GCaMP6s mice (*N* = 5), which permits visualization of mural cell–free Ca^2+^ using the PDGFR-β promoter (35). The MES elicited similar postictal hypoxia measures (Figure 1, E–G) across both Cre-lox cross lines and compared with C57BL/6J mice. These data show that MES induces reliable seizures and that postictal hypoxia generalizes across sexes and different mouse lines.

### Seizures cause sustained, COX-2–sensitive arteriole constriction along with astrocytic and vascular smooth muscle Ca^2+^ recruitment.

**Figure 2:**
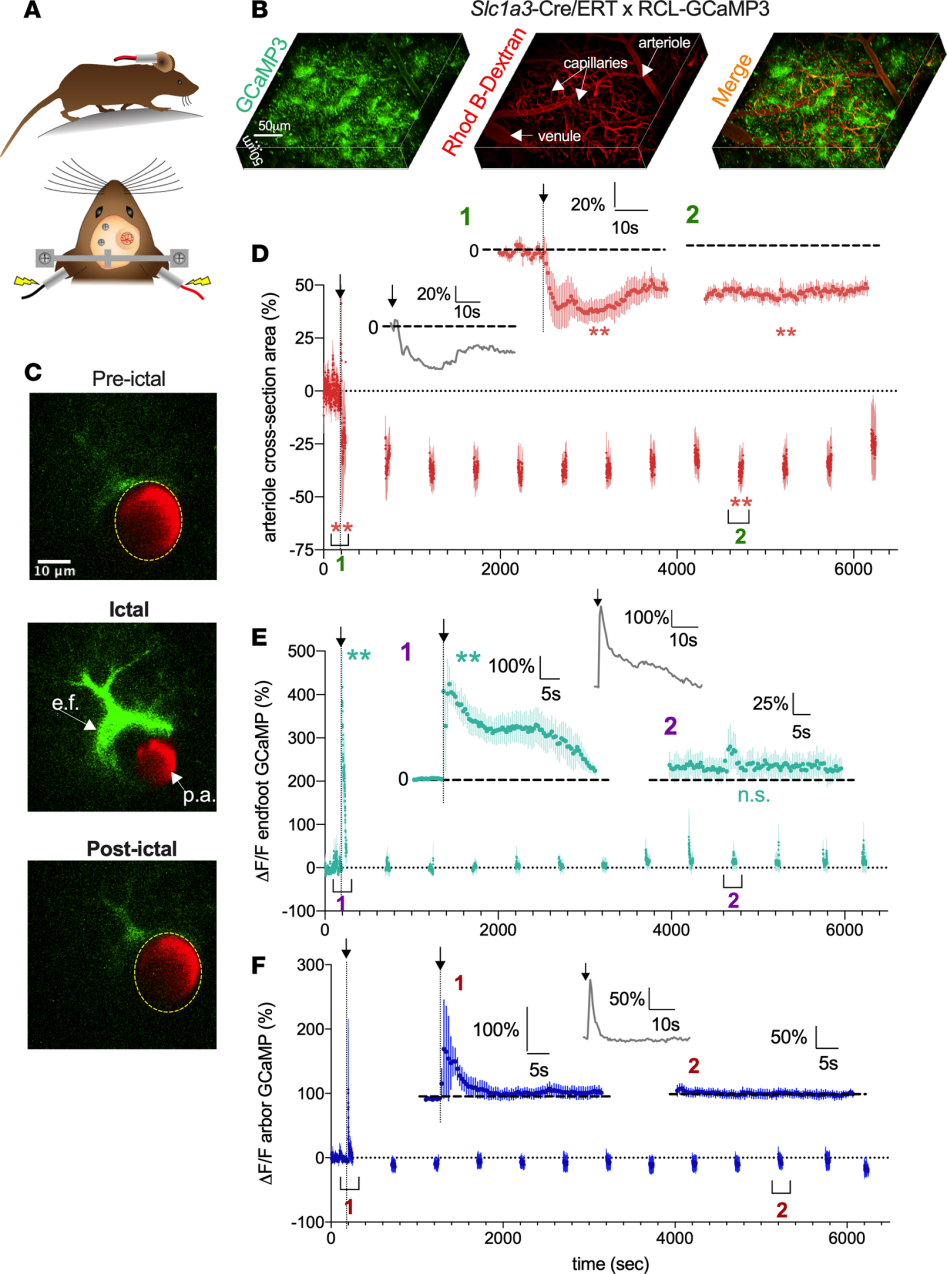
Seizure-induced sustained arteriole constriction is associated with an initial transient rise in astrocytic endfoot Ca^2+^. (**A**) Schematics of awake-mouse experimental setup. (**B**) Reconstruction in 3D of the superficial barrel cortex from a *Slc1a3*-Cre/ERT RCL-GCaMP3 mouse. Astrocytes expressing GCaMP3 are shown in green; the vasculature is loaded with Rhod B-dextran shown in red. (**C**) Cross section of a penetrating arteriole (p.a.) enwrapped by an endfoot (e.f.). Images show preictal (top), severe vasoconstriction and a large astrocyte Ca^2+^ rise triggered by MES during the ictal period (middle), and the postictal period (bottom). (**D**). Summary time course of arteriolar diameter in *Slc1a3*-Cre/ERT RCL-GCaMP3 mice (*N* = 5). To limit photobleaching and/or photodamage, measurements were taken for 60 seconds every 300 seconds. Arrow and vertical dotted line indicate MES (0.2 second). *Inset:* Temporal close-up of percent diameter changes during the ictal (*t* test, t[4] = 8.06, *P* < 0.001) and postictal (*t* test, t[4] = 4.04, *P* = 0.007) period, and representative trace of diameter response to 0.2-second MES. (**E**) Summary time course of endfoot Ca^2+^ measurements in the same experiment as diameter measures (*N* = 4). Vertical dotted line indicates MES (0.2 second). *Inset:* Temporal close-up of endfoot Ca^2+^ response during the ictal (*t* test, t[3] = 6.36, *P* = 0.007) and postictal (*t* test, t[3] = 1.63, *P* = 0.20) period and representative trace of endfoot Ca^2+^ to 0.2-second MES. (**F**) Summary time course of astrocyte arbor Ca^2+^ measurements in the same experiment as diameter measures (*N* = 4). Vertical dotted line indicates MES (0.2 second). *Inset:* Temporal close-up of astrocyte arbor Ca^2+^ response during the ictal (*t* test, t[3] = 3.43, *P* = 0.04) and postictal (*t* test, t[3] = 1.56, *P* = 0.2) period and representative trace of astrocyte arbor Ca^2+^ to 0.2-second MES. Data represent mean ± SEM. *P < 0.05, **P < 0.01, ***P < 0.001.

Severe cerebral hypoxic events are often the consequence of inadequate blood flow, which can occur via several mechanisms, including sustained pathological vasoconstriction of contractile microvasculature, thrombotic or hemorrhagic stroke, and a precipitous drop in perfusion pressure. Therefore, determining how local cerebral oxygenation and perfusion become dramatically decreased after epileptiform activity can identify new routes for intervention. We used 2-photon fluorescence microscopy in awake, head-restrained mice trained to run a floating spherical treadmill (25, 26). We imaged single penetrating arterioles labeled with rhodamine B isothiocyanate–dextran (Rhod B–dextran) in the barrel cortex to explore cellular Ca^2+^ signals associated with neocortical hypoperfusion/hypoxia. We first used *Slc1a3*-Cre/ERT × RCL-GCaMP3 mice to observe astrocyte Ca^2+^ changes, along with microvascular responses, before, during, and after MES-induced seizure (Figure 2, A and B). In response to 0.2 seconds of MES, arterioles displayed a robust, prolonged vasoconstriction (ictal: –51.4% ± 8.5%, *P* < 0.001; postictal at time 4800 seconds: –37.7% ± 4.3%, *P* = 0.007, *N* = 5, Figure 2, C and D) (other postictal time points displayed similar statistical outcomes). The vasoconstriction lasted more than 90 minutes in some trials, exhibiting a similar temporal profile to the deterioration and eventual recovery of oxygen levels. Even at peak constriction, the arteriole lumen could still be clearly visualized, and RBC movement detected, suggesting the vessel did not occlude. Examining astrocyte Ca^2+^ along with vascular lumen dynamics during and after seizure, we found that MES caused a rapid elevation in astrocyte endfoot Ca^2+^ during the initiation of vasoconstriction (389.4% ± 81.4%, *P* = 0.007, *N* = 4, Figure 2, C and E). The endfoot Ca^2+^ elevation decayed to a plateau (180.5% ± 72.6%, Figure 2, C and E) for approximately 20 seconds before returning to baseline values, whereas vasoconstriction was sustained. Postictally at 4800 seconds, endfoot Ca^2+^ was not different from baseline values (15.1% ± 13.5%, *P* = 0.2, *N* = 4, Figure 2, C and E) (other postictal time points displayed similar statistical outcomes), whereas lumen diameter was significantly smaller. We also examined Ca^2+^ in the astrocyte arbor, which is replete with perisynaptic fine processes that detect neural activity. The astrocyte arbor displayed a similar rapid elevation in Ca^2+^ to MES (61.02% ± 17.81%, *N* = 4, *P* = 0.04, Figure 2F); however, unlike the endfoot that reached a secondary plateau, arbor Ca^2+^ rapidly returned to baseline. Postictally, astrocyte arbor Ca^2+^ was not different from baseline (–5.3% ± 8.8% at 5200 seconds, *N* = 4, *P* = 0.5, Figure 2F).

**Figure 3:**
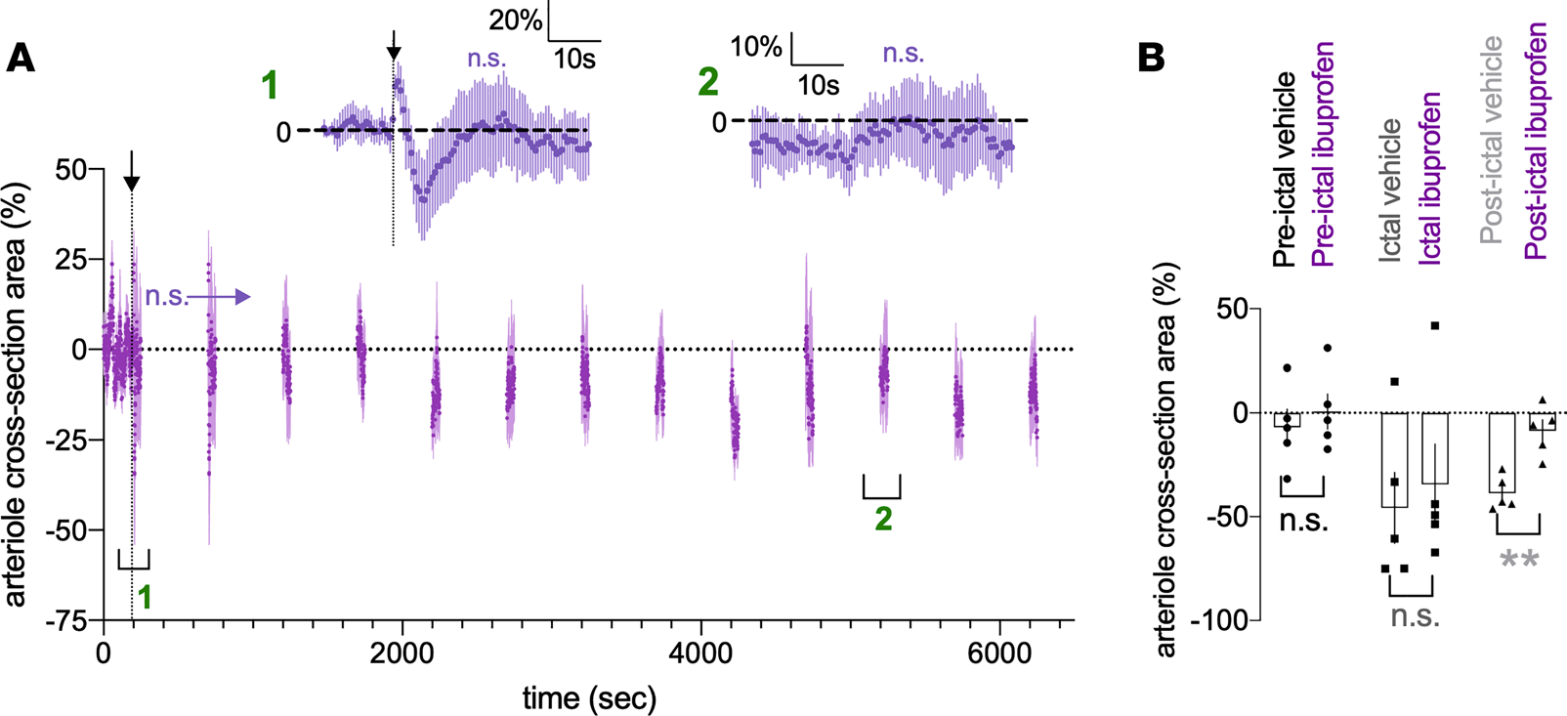
Prolonged postictal vasoconstriction is prevented by COX-2 antagonism. (**A**) Summary time course of arteriolar diameter in the presence of ibuprofen in response to MES (*N* = 5). To limit photobleaching and/or photodamage, measurements were taken for 60 seconds every 300 seconds. Arrow and vertical dotted line indicate MES (0.2 second). *Inset:* Temporal close-up of percent diameter changes during the ictal and postictal period (5200 seconds vs. baseline: *t* test, t[4] = 0.92, *P* = 0.41). (**B**) Summary data comparing vehicle i.p. injection (gray) with ibuprofen (purple), at a preictal (*t* test, t[4] = 1.31, *P* = 0.25), ictal (*t* test, t[4] = 0.38, *P* = 0.72), and postictal time point (5200 seconds: *t* test, t[4] = 4.14, *P* = 0.007). Data represent mean ± SEM. **P < 0.01.

Seizure-induced hypoxia is blocked by COX-2 antagonism as well as with COX-2–KO animals (6). To test for the involvement of COX-2 in our MES, awake-mouse, 2-photon model, we used ibuprofen (100 mg/kg) to determine whether sustained vasoconstriction could be reduced. Because ibuprofen is ineffective if administered shortly after seizure (6), indicating the necessity of ecosanoid production during epileptiform activity, we pretreated mice i.p. 30 minutes before imaging; any efficacy of ibuprofen could be readily translatable to humans with recurrent seizures. Notably, in the presence of ibuprofen, there was a brief vasodilation immediately following MES lasting approximately 2 seconds not observed in vehicle-treated animals (Figure 3A). Further, ibuprofen failed to block the initial rapid constriction after MES within the first 10 seconds (ictal control: –45.8% ± 17.0% vs. ictal ibuprofen: –34.7% ± 19.4%, *P* = 0.72, Figure 3A). However, COX-2 antagonism completely prevented the development of the prolonged vasoconstriction when compared with vehicle control (postictal vehicle at 5200 seconds: –38.7% ± 3.6% vs. postictal ibuprofen at 5200 seconds: –8.5% ± 5.3%, *P* = 0.007, *N* = 5, Figure 3, A and B) (other postictal time points displayed similar statistical outcomes). These data provide evidence that postictal hypoperfusion/hypoxia occurs via a COX-2–dependent, enduring vasoconstriction in local microvasculature.

**Figure 4:**
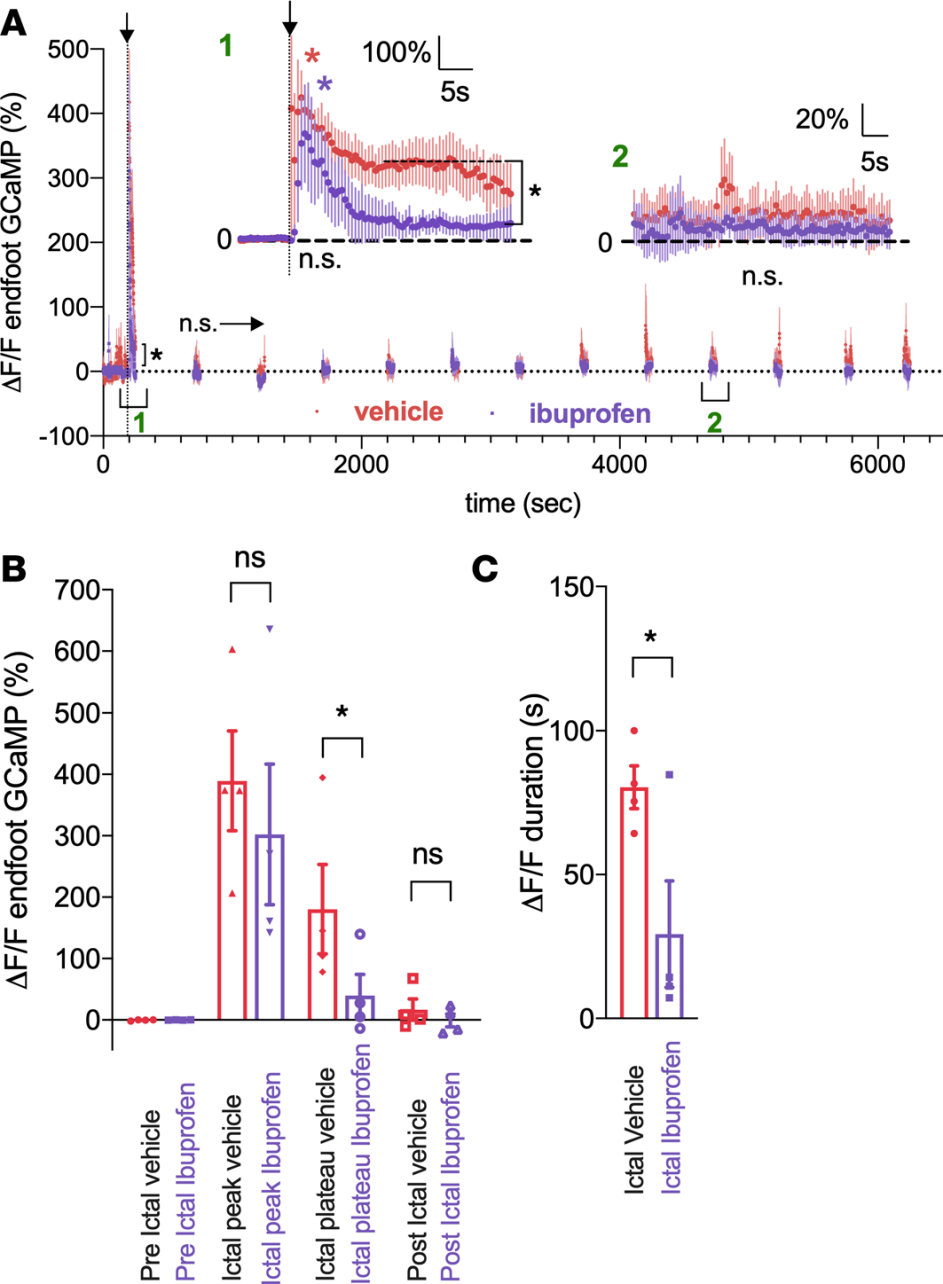
COX-2 antagonism affects the secondary component of the MES evoked endfoot Ca^2+^ signal. (**A**) Summary time course of endfoot Ca^2+^ in the absence (red) or presence (purple) of ibuprofen in response to MES (*N* = 5). To limit photobleaching and/or photodamage, measurements were taken for 60 seconds every 300 seconds. Arrow and vertical dotted line indicate MES (0.2 second). *Inset:* Temporal close-up of percentage endfoot Ca^2+^ changes during the ictal and postictal period (5200 seconds) comparing vehicle (red) versus ibuprofen (purple). (**B**) Summary data comparing vehicle i.p. injection (red) with ibuprofen (purple) at a preictal (*t* test, t[3] = 0.99, *P* = 0.4), ictal peak (*t* test, t[3] = 1.57, *P* = 0.21), ictal plateau (*t* test, t[3] = 3.31, *P* = 0.04), and postictal time point (5200 seconds: [*t* test, t{3} = 0.77, *P* = 0.5]). (**C**) Summary data comparing the duration of the endfoot Ca^2+^ signal in vehicle (red) and ibuprofen (purple) during the ictal period (*t* test, t[3] = 3.9, *P* = 0.03). Data represent mean ± SEM. *P < 0.05.

Next, we examined the effect of ibuprofen on astrocyte Ca^2+^ dynamics in response to MES. Ibuprofen did not prevent the immediate, large elevation in astrocyte endfoot Ca^2+^ to MES (vehicle: 389.4% ± 81.4%; ibuprofen: 302.3% ± 114.6%, *N* = 4, *P* = 0.21, Figure 4, A and B), yet ibuprofen prevented the secondary plateau Ca^2+^ elevation in endfeet (vehicle: 180.5% ± 72.6%; ibuprofen: 39.7% ± 34.3%, *N* = 4, *P* = 0.01, Figure 4, A and B), shortening the duration of the signal (vehicle: 80 ± 7.5 seconds vs. ibuprofen 29 ± 9 seconds, *N* = 4, *P* = 0.03, Figure 4C). We found no difference in endfoot Ca^2+^ postictally at 5200 seconds with COX-2 antagonism (vehicle: 17.0% ± 17%; ibuprofen: –0.7% ± 10.6%, *N* = 4, *P* = 0.5, Figure 4, A and B). These data demonstrate 2 components exist for endfoot Ca^2+^ elevation to MES: (a) an early signal that is independent of COX-2 activity and (b) a later, lower amplitude signal that is either directly dependent on COX-2, such as through the activation of activation of E-type prostanoid (EP) receptors on endfeet, or indirectly dependent on COX-2 activity, such as via constriction-evoked endfoot Ca^2+^ signals (see Discussion for additional details).

**Figure 5:**
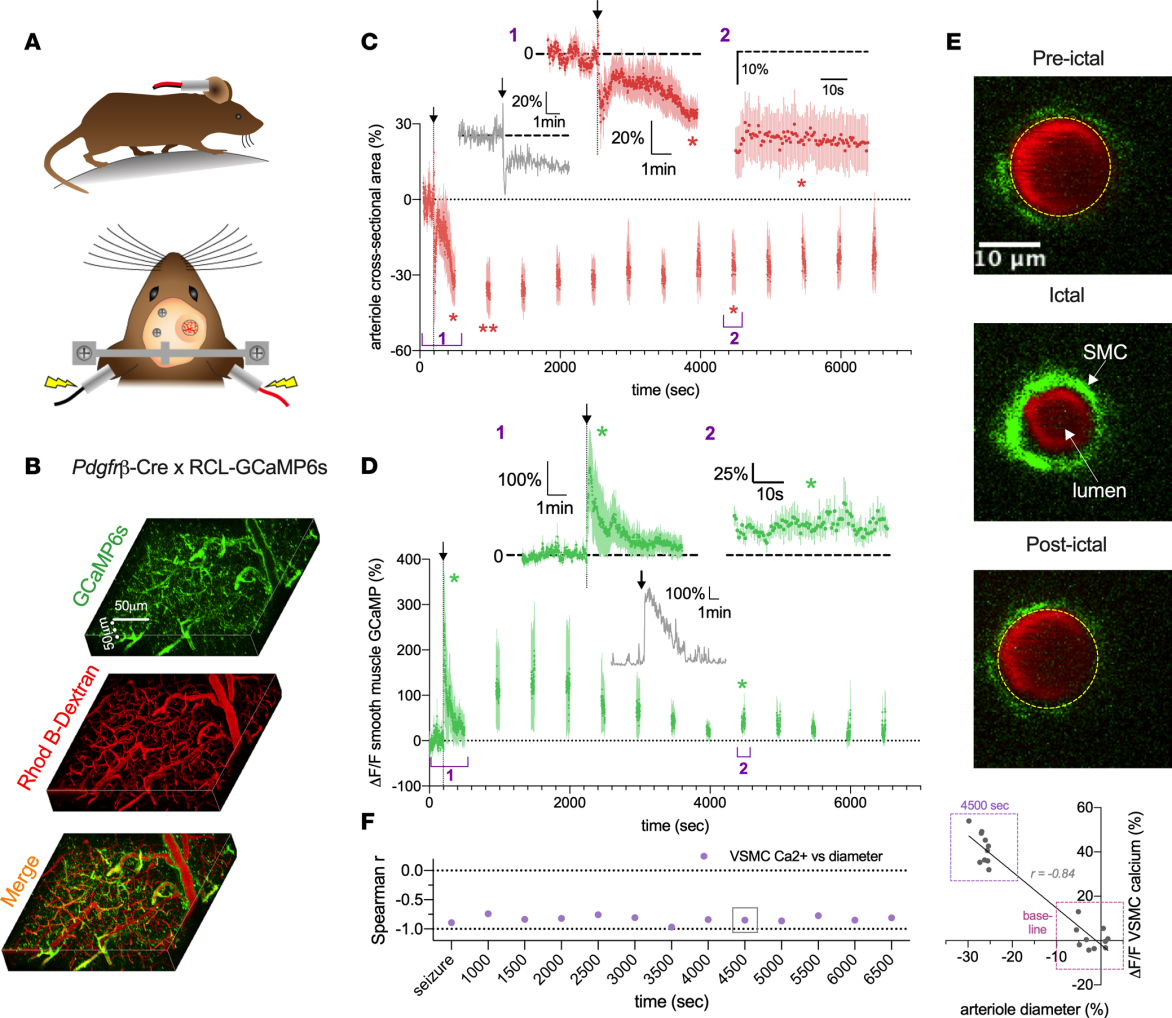
Seizure-induced sustained arteriole constriction is associated with rapid and prolonged vascular smooth muscle cell Ca^2+^ elevation. (**A**) Schematics of awake-mouse experimental setup. (**B**) Reconstruction in 3D of the superficial barrel cortex from a *PdgfrB*-Cre RCL-GCaMP6s mouse. VSMC expressing GCaMP6s are shown in green; the vasculature loaded with Rhod B-dextran is shown in red. (**C**) Summary time course of arteriole diameter responses (*N* = 5). Arrow and vertical dotted line indicate MES (0.2 second). To limit photobleaching and/or photodamage, measurements were taken for 60 seconds every 300 seconds. *Inset:* Temporal close-up of percent diameter changes during the ictal (*t* test, t[4] = 3.74, *P* = 0.02) and postictal (*t* test, t[4] = 3.58, *P* = 0.023) period and representative trace of diameter in response to 0.2-second MES. (**D**) Summary time course of VSMC Ca^2+^ elevations in the same experiments as diameter measures. *Inset:* Temporal close-up of percentage VSMC Ca^2+^ changes during the ictal (*t* test, t[4] = 2.42, *P* = 0.036) and postictal (*t* test, t[4] = 3.03, *P* = 0.038) period. *Inset:* representative trace of VSMC Ca^2+^ in response to 0.2-second MES. (**E**) Cross section of a penetrating arteriole (red) with VSMC expressing GCaMP6s (green). Images show baseline (top), the ictal period (middle), and the postictal period (bottom). (**F**) Left: Summary of calculated Spearman’s *r* values between changes in arteriole diameter and VSMC Ca^2+^ during the postictal period. Right: Correlation between VSMC Ca^2+^ and arteriole diameter during baseline (100 seconds before MES) and postictal period (4500 seconds). Each data point represents a 10-second bin and averages across all 5 animals. Data represent mean ± SEM. *P < 0.05, **P < 0.01.

Finally, we examined mural cell Ca^2+^ activity by using *PdgfrB*-Cre × RCL-GCaMP6s mice (*N* = 5, Figure 5, A and B). By observing penetrating arterioles in response to MES, we found that VSMC Ca^2+^ exhibited a rapid increase in free Ca^2+^ (257.7% ± 108.9%, *P* = 0.036, Figure 5, D and E) as the vessel constricted (–36.5% ± 7.6%, *P* = 0.02, Figure 5, C and E), yet the Ca^2+^ signal maintained elevated above baseline values at various (but not all) time points, even up to 4500 seconds (42.7% ± 15.4%, *P* = 0.038) during the sustained vasoconstriction (–24.0% ± 7.6%, *P* = 0.023). Further, we examined the Spearman’s correlation coefficient, *r*, between vascular smooth muscle Ca^2+^ and diameter using the averaged data from all 5 mice between baseline data compared with each ictal and postictal imaging epoch (each 30 seconds of data) and found significant negative relationships (4500 seconds: *r* = –0.84, *P* < 0.001, Figure 5F). This analysis suggested that VSMC Ca^2+^ corresponded to the changes in arteriole diameter. Collectively, these data revealed the temporal dynamics of penetrating arteriole diameter, endfoot Ca^2+^, and VSMC Ca^2+^ during and up to 90 minutes after seizure in awake mice.

## Discussion

Here, we observed the calcium dynamics in astrocytes and VSMCs after brief, maximum electroconvulsive seizures along with corresponding changes in penetrating arteriole diameter. For the first time to our knowledge, we captured COX-2–sensitive, severe vasoconstriction in cerebral microvasculature in vivo that was sustained for more than 1 hour after the ictal event. Astrocyte endfeet and vascular smooth muscle calcium signals were distinctly different in their temporal profile, with endfoot calcium restricted largely to the ictal period, whereas smooth muscle calcium elevation was variable and lasted up to 75 minutes after MES. Interestingly, the late component of the endfoot Ca^2+^ elevation to MES was also sensitive to COX-2 blockade. Finally, we showed that postictal hypoxia generalizes equally to male and female mice and to the Cre-lox knockin crosses used in our study.

COX-2 plays a central role in coordinating a cascade of events to induce severe hypoxia following electrographic seizures (6). Here, we show that COX-2 is necessary for pathological vasoconstrictions, which is most likely the primary cause for the hypoxia/hypoperfusion event. Activity-dependent induction of COX-2 in neurons plays an important role in normal neurovascular coupling by producing vasoactive prostanoids that act on blood vessel receptors to cause vasodilation (36–38). Under pathophysiological activation during electrographic seizures, we observed prolonged vasoconstriction rather than transient vasodilation. However, in the presence of ibuprofen, a fast vasodilation was revealed immediately following MES. Further, ibuprofen failed to block an ensuing short-lived vasoconstriction, suggesting these events involved a different enzyme and messenger system than that of COX-2, which was involved in the enduring pathological vasoconstriction. Our analysis of endfoot Ca^2+^ in ibuprofen revealed an interesting reduction in only the secondary plateau Ca^2+^ signal, leaving the early increase in Ca^2+^ intact. This signal could be attributed to the generation of eicosanoids and subsequent binding to EP receptors expressed on endfeet, the activation of which elevates Ca^2+^ (39). EPR1 transcripts are found in cortical astrocytes (40). Alternatively, this secondary endfoot Ca^2+^ signal could be evoked by the vasoconstriction itself, via endfoot stretch sensing (41). Here, the Ca^2+^ signal would be lost indirectly through the loss of vasoconstriction when COX-2 is blocked. The latter idea is tractable because we failed to observe the secondary plateau increase in Ca^2+^ in the astrocyte arbor, which would not be stretched to the same degree as perivascular endfeet. In addition, if the secondary Ca^2+^ signal was the result of neuronally derived prostaglandins, one might expect this signal to be present in perisynaptic processes, which it was not. However, arguing against this idea is that the plateau in endfoot Ca^2+^ was not sustained for the entire postictal vasoconstriction and lasted only for 20–30 seconds.

Although astrocytes have low levels of COX-2 under basal conditions, these cells upregulate COX-2 in models of epilepsy (42). Thus, the source of the constricting eicosanoids still needs to be delineated with cell type–selective COX-2–KO experiments. For example, whereas seizure activity is expected to cause large increases in free calcium in principal neurons (43), we observed a large calcium signal also in endfeet, which may have produced COX-dependent vasoconstrictors. Prostanoids such as prostaglandin E_2_ that derive from COX activity can produce vasoconstriction if the appropriate EP receptors (EP1 and EP3) are present on vasculature (44, 45). Our results, therefore, indicate long-term changes to vascular smooth muscle calcium. Transient release of constrictor agents from parenchymal cells is all that is required, as our previous data showed that COX-2 antagonism after seizure was ineffective, whereas postictal administration of an L-type antagonist was effective at blocking the sustained drop in tissue oxygen (6). The way in which vascular calcium elevation becomes sustained remains unclear; however, a mechanism that acts to facilitate L-type Ca^2+^ channel opening and/or decrease plasmalemma K^+^ channel opening, which help set resting membrane potential, thereby affecting L-type recruitment, are possibilities. Therefore, our observations should assist future studies investigating these ideas.

We previously used acute hippocampal slices and observed the diameter of local arterioles (6). The conditions in slices differ substantially from in vivo conditions, especially regarding the lack of blood pressure and flow, and this could potentially misrepresent the arteriolar results. For example, slices do not capture vascular temporal dynamics accurately, nor is the magnitude of the arteriole diameter measurements comparable to in vivo (46). However, the degree of constriction observed in slices provided estimates of the changes in blood perfusion that were observed with laser Doppler flowmetry. In addition, the vasoconstriction following seizure stimulation in slices was sensitive to acetaminophen and nifedipine, which supports this constriction follows the same set of mechanisms that occur in vivo. Notably, our in vivo observations of arterioles were essential to either support or refute our current model.

Postictal behavioral impairments last much longer than the seizures themselves and negatively affect quality of life (47). The mechanism underlying these disruptions was previously unknown, and no treatments for the postictal state are in clinical practice. Our discovery that brief, mild seizures lead to an extended vasoconstrictive event is critically important because it establishes that seizures could injure the brain through postictal hypoperfusion/hypoxia, and not necessarily through the seizure itself. Severe hypoxia may be an important component of seizure-induced brain damage (48), and since postictal hypoperfusion/hypoxia is COX-2 dependent, this hypothesis can be assessed. Although controversy exists (49), it is generally accepted that COX-2 inhibition is neuroprotective from seizures. Further, with genetic or pharmacological COX-2 inhibition, seizure-induced brain damage can be dramatically reduced (50–52). Although at the time of these studies researchers were unaware of postictal hypoperfusion/hypoxia and the requirement of COX-2 in this response, they provided support for the hypothesis that postictal hypoperfusion/hypoxia injures the brain (8). Given the central role of brain injury in epileptogenesis (53), preventing injury from vasoconstriction-induced hypoperfusion/hypoxia may be a novel preventative treatment strategy in epilepsy.

## Methods

### Mice.

All studies were either performed on young adult male and female C57BL/6J (The Jackson Laboratory 000664), *Slc1a3*-Cre/ERT (The Jackson Laboratory 012586) × RCL-GCaMP3 (The Jackson Laboratory 014538, Ai38), and *PdgfrB*-Cre × RCL-GCaMP6s (The Jackson Laboratory 024106, Ai96) mice weighing between 21 and 30 g. *PdgfrB*-Cre mice were provided by Volkhard Lindner (Maine Medical Research Institute, Scarborough, Maine, USA) and Andy Shih (Seattle Children’s Institute, Seattle, Washington, USA). Mice were housed individually in clear plastic cages and maintained on a 12-hour light/12-hour dark cycle, with lights on at 7 a.m., in separate colony rooms under specified pathogen–free conditions. Food and water were available ad libitum. All experimental procedures occurred during the light phase.

### Eliciting and recording seizures and oxygen detection.

Electrodes were constructed from Teflon-coated, stainless steel wire, 178 μm in diameter (A-M Systems). Wire ends were stripped of Teflon and connected to gold-plated male amphenol pins. Mice were anesthetized with a 4% isoflurane and maintained between 1% and 2%. Lidocaine (2%) was administered subcutaneously at the incision site. One bipolar electrode was chronically implanted under stereotaxic control in barrel cortex at –1.75 AP, +3 ML, –1.5 DV with an oxygen-sensing probe positioned nearby at –1.65 AP, +3.5 ML, –1.5 DV relative to bregma. The implants were adhered and anchored to the skull using dental cement and a ground electrode. Subsequent experimental procedures commenced no earlier than 5 days following surgery.

Oxygen recordings were obtained using an implantable fiber-optic oxygen-sensing device. A total of 525 nm light pulses induce fluorescence (measured at 650 nm) at the platinum tip that is quenched by oxygen within a local area (~500 × 500 × 500 µm) and uses the fluorescence decay time to derive pO_2_ (54). The technology (Oxylite, Oxford Optronics) does not consume oxygen while measuring absolute pO_2_ values. The manufacturer individually calibrates each biologically inert probe, called an optode. The implant is inserted under isoflurane anesthesia, and every effort was made to minimize suffering. We allowed at least 7 days between implantation and initiation of measurements to ensure that the effects of acute trauma were minimized. pO_2_ measurements at 1 Hz can then be made at any time by connecting the implant to the Oxylite using an extension fiber-optic lead. The probe provides accurate and continuous measurements of local pO_2_ levels in brain tissue in awake, freely moving animals over several weeks and is exquisitely sensitive to oxygen perturbations in relation to epileptiform activity (55).

On test days, mice were connected to the EEG, oxygen-sensing system, and saline-soaked ear clips and allowed 5 minutes to adjust before any measurements were taken. A seizure was elicited after 100 seconds of baseline recording using a suprathreshold MES stimulus, which was delivered through the ear clips via a GSC 700 shock generator (model E1100DA) (Grason-Stadler). Oxygen levels were recorded before and after the delivery of a 0.2-second train of 60 Hz biphasic sine wave pulses (27). Seizure duration was recorded by observing seizure behavior (56). Once the EEG returned to baseline following a seizure, the electrodes were disconnected, but the fiber-optic cable for oxygen-sensing was left attached. The fiber-optic cable was disconnected when pO_2_ levels returned to baseline.

### Awake in vivo 2-photon preparation.

All surgeries followed the procedures as previously described (25). Briefly, 1 week before the imaging session, a custom head bar was surgically installed without performing a craniectomy. The mouse was then returned to a new home cage to recover for 2 days (single housing). The mouse was then trained on a passive air-supported Styrofoam ball treadmill with its head restrained. The training was conducted on 2 consecutive days for 30 minutes each day. The mice were habituated to the seizure setup without inducing the actual seizure (i.e., ears were clipped as above), then returned to their home cage after each training session. On the second day, the mouse received MES during ball training. On the imaging day, a cranial window was created as previously described over the primary somatosensory cortex, with both bone and dura removed.

### Vessel indicators.

Rhod B–dextran (MW 70,000, MilliporeSigma) was tail vein injected (100–200 mL of 2.3% w/v solution in saline solution) to visualize the blood plasma. To image and quantify arteriole changes, we quantified changes in lumen cross-sectional area in every frame using particle analysis in ImageJ (NIH). Before imaging, the mouse recovered on the treadmill with its head immobilized for 30 minutes.

### Two-photon fluorescence microscopy.

Fluorescence images were obtained using a custom-built in vivo 2-photon microscope (57) fed by a tunable Ti:Sapphire laser (Chameleon, Coherent, Ultra II, ~4 Wavg power, 670–1080 nm, ~80 MHz, 140 fs pulse width), equipped with GaAsP photomultiplier tubes (Hamamatsu) and controlled by open-source ScanImage software (https://wiki.janelia.org/). We used a Nikon ×16, 0.8 numerical aperture (NA), 3 mm working distance (WD) objective lens or a ZEISS ×40, 1.0 NA, 2.5 mm WD objective lens. GCaMP3 and GCaMP6s were excited at 920 nm. Green fluorescence signals were filtered using a 525/50 nm bandpass (BP), and orange/red light was filtered using a 605/70 nm BP (Chroma Technology). Bidirectional *xy* raster scanning was used at a frame rate of 0.98 Hz. Typically, 1 image frame was corrupted from the movement artifact associated with MES stimulation; thus 1 second was lost from the data set, which was inconsequential given the time course of the events measured. Changes in astrocytes and VSMC free Ca^2+^ were calculated as ΔF/F = ([F_1_ – F_0_]/F_0_) × 100, where F is fluorescence, 1 is at any given time point, and 0 is an average baseline value.

### Behavior capture.

Near-infrared LED (780 nm) and camera were used to capture simple behaviors, such as resting, running, whisking, and tracking responding to MES concurrently with 2-photon fluorescence imaging for all experimental trials at a frame rate of 14 Hz.

### Seizure induction for 2-photon imaging.

On an imaging day, mice were connected to the ear clips and allowed 5 minutes before imaging. A seizure was elicited after 5 minutes of baseline imaging using a suprathreshold MES stimulus that was delivered through the ear clips via a GSC 700 shock generator (model E1100DA) (Grason-Stadler). Continuous imaging was conducted for 15 minutes and 1 minute for every 5 minutes afterward to reduce photobleaching/photodamage.

### Replicates.

For technical replicates (a test performed on the same sample multiple times), whether recording oxygen and EEG or performing 2-photon imaging, each animal received 3 MES total. For biological replicates (a test performed on biologically distinct samples representing an identical time point or treatment dose), we performed oxygen and EEG measurements on 4 distinct groups of mice, male C57BL/6J mice, female C57BL/6J mice, astrocyte Ca^2+^ reporter mice, and VSMC Ca^2+^ reporter mice, and observed the same postictal hypoxia in all groups. We also performed the same 2-photon imaging procedure on 2 groups, astrocyte Ca^2+^ reporter mice and VSMC Ca^2+^ reporter mice, and observed the same postictal vasoconstriction in both groups. Mice that did not exhibit behavioral seizures following stimulation in the 2-photon imaging experiments (where there were no electrographic measures) were excluded from the study. Additionally, outliers were not excluded. Given the large percentage change in brain oxygenation after seizure from our previous studies, 5 animals per group were selected for the sample size here. One mouse in the GLAST-GCaMP3 group failed to exhibit GCaMP3 expression; therefore, becoming *N* = 4, but vasculature could still be measured. One penetrating arteriole was examined per mouse, tracked at a single focal plane. Mice were obtained from our in-house breeding colonies, and each served as its own control.

### Statistics.

All statistical analyses were performed using Prism version 5.01 (GraphPad). Statistical *N* represented the measurements from a given mouse. Two-tailed paired or unpaired (as appropriate) parametric *t* tests were used for experiments with only 2 groups or 1 comparison. One-tailed *t* tests were used for ibuprofen versus vehicle on arteriole diameter measures, as well as VSMC Ca^2+^ changes versus baseline, as our previous published data allowed us to predict the direction of the effect. A 1-way ANOVA was used for comparisons of more than 2 groups with a follow-up Tukey’s test. Repeated measures statistics were used for all analyses within subject experiments. Spearman’s correlation coefficient, *r*, was used to look at the relationship between VSMC Ca^2+^ and arteriole diameter. The following *P* values were deemed significant: *P* < 0.05, *P* < 0.01.

### Study approval.

Mice were handled and maintained according to the Canadian Council for Animal Care guidelines. These procedures were approved by the Life and Environmental Sciences Animal Care and Health Sciences Animal Care Committees at the University of Calgary (AC15-0133 and AC16-0272).

## Author contributions

GCT and GRG conceptualized the study. CHTT and AGG performed the investigations. GRG and GCT supervised the entire project. All authors contributed to the writing of the original draft and subsequent versions as well as the editing and review of the final manuscript.

## Version 1 10/02/2020

Electronic publication
